# The relationship between HBV serum markers and the clinicopathological characteristics of hepatitis B virus-associated glomerulonephritis (HBV-GN) in the northeastern chinese population

**DOI:** 10.1186/1743-422X-9-200

**Published:** 2012-09-14

**Authors:** Lei Zhang, Hongxue Meng, Xingying Han, Changsong Han, Chuanhui Sun, Fei Ye, Xiaoming Jin

**Affiliations:** 1 Department of Pathology, Basic Medical Science College, Harbin Medical University, 157 Baojian Road, Nangang District, Harbin, 150081, China

**Keywords:** HBV serum markers, Pathological subtype of hepatitis B virus-associated glomerulonephritis, Renal functional parameters, Clinical manifestations or symptoms

## Abstract

**Background:**

To investigate the effect of HBV markers on HBV-GN.

**Methods:**

The immunohistochemistry was used to detect HBsAg and HBcAg in frozen sections of renal biopsy, the changes in HBV serum markers, renal functional parameters and clinical manifestations or symptoms were observed to analyze renal damage.

**Results:**

Using renal biopsy data from 329 cases, this study found that the most common pathological subtype in HBV-GN was mesangioproliferative glomerulonephritis (MsPGN) (24.9%, *P* <0.05), and 29.4% of patients who show serological HBsAg, HBeAg and anti-HBc positive developed membranoproliferative glomerulonephritis (MPGN) (*P* <0.05). The immunohistochemistry was used to detect HBsAg and HBcAg in frozen sections.50% of HBsAg and HBcAg deposits was observed in the glomeruli of MPGN patients, while 36.6% of HBsAg and 43.9% of HBcAg deposited in the glomeruli of MsPGN patients. The deposits of HBsAg and HBcAg in glomeruli were directly correlated with IgA, IgG, IgM and C3 deposits. In addition, cases with a moderate to severe decrease as reflected by the glomerular filtration rate (GFR) were predominantly patients with MPGN (31.6%, *P* <0.05) or MsPGN (21.1%, *P* <0.05). Patients who were serological HBsAg, HBeAg and anti-HBc positive or HBsAg, anti-HBe and anti-HBc positive mainly exhibited urine and renal parameter changes.

**Conclusion:**

Examination of HBV markers in serum and renal biopsy will be useful for clinicians to predict the renal damage in early stage when it is reversible in HBV-GN.

## Background

Hepatitis B virus (HBV) infection occurs worldwide, with a high prevalence in most developing countries in Southeast Asia and Africa. Extra-hepatic manifestations of HBV infection are being increasingly recognized, with an expanding prevalence. One of the most common manifestations is HBV-GN [1,2]. Different pathological types of glomerular lesions have been described in association with HBV infection, including pathological patterns such as MPGN, MsPGN, and Membranoproliferative glomerulonephritis (MN). MPGN is a chronic progressive glomerulonephritis that is common in young and middle-aged adults, and is characterized by an increase in mesangial cellularity and matrix, with thickening of glomerular capillary walls secondary to subendothelial and mesangial deposition of circulating immune complexes and/or complement factors, cellular entrapment, and new basement membrane formation. It is always present in nephrotic syndrome or acute nephritic syndrome [3]. MPGN has a poorer prognosis than MN and is associated with a high risk of renal failure. MsPGN is common in teenagers and demonstrates mesangial hypercellularity and/or increase in mesangial matrix [4]. Its clinical manifestation and prognosis of moderate-severe MsPGN are similar to those of MPGN. MN is a chronic progressive glomerulonephritis that is common in adults, characterized by sub-epithelial immune deposits inducing non-selective proteinuria. However, among these histological types, MN has been reported as the commonest pathological type of HBV-GN in Hong Kong and South Africa [5-7], compared to IgA nephropathy followed by MN in Thailand [8] and MN and MPGN in Japanese adults [9].

HBV infection is prevalent in China, and HBV-GN commonly causes renal damage secondary to HBV infection. At present, there are no definite diagnostic criteria for HBV-GN. However, the reference diagnostic criteria used in China are as follows [10]: a) HBsAg-positive serum; b) presence of glomerular nephritis, excluding lupus nephritis and other secondary glomerular diseases; and c) HBV antigens including HBsAg or HBcAg, or HBV DNA measured by PCR in nephridial tissue. Among these criteria, the third criterion is required for a final diagnosis. In our study, for serum HBsAg-positive patients, including HBsAg-, HBeAg- and anti-HBc-positive patients; HBsAg-, anti-HBe- and anti-HBc-positive patients; HBsAg- and anti-HBc-positive patients; and simple HBsAg-positive patients, it is of great clinical significance to analyse the degree of HBV antigen deposited in renal tissue and the glomerular impairment. These different serum markers of HBV are an indirectly measure of the injury and prognosis of HBV-GN. The present study summarised and analysed the changes in HBV serum markers, renal functional parameters, clinical manifestations or symptoms and the pathological data and of 329 clinical cases of HBV-GN from Harbin in northeastern China.

## Methods

### The clinical data

A total of 329 cases from among 5250 patients (329/5250, 6.27%), who underwent renal biopsies and were diagnosed as HBV-GN with HBsAg-positive serum, were analysed in this study. Diagnoses were made by the Department of Pathology, Harbin Medical University, from January 1998 to April 2010. There were 221 males and 108 females, with a male-to-female ratio of 2.05:1. All patients were between 17 years old and 63 years old with, an average age of 33.28 ± 9.45 years. 3136 cases from 5250 patients (3136/5250, 59.7%) with HBsAg-negative serum were taken as negative control. Negative control was selected and matched for gender and age distribution (average age of 33 ± 14 years and male-to-female ratio of 1.97:1).

### The pathological classification and the diagnosis criteria

The lesions of the 329 cases of glomerular nephritis were classified according to the 1990 WHO Classification criteria [11]. The diagnosis of nephropathy was confirmed by pathology. Sections from all biopsy specimens also were stained routinely with hematoxylin and eosin (H&E), periodic acid-sliver methenamine (PASM), Masson’s trichrome, and antibodies against IgA, IgG, IgM and the C3 complement component. Fluorescently labelled goat anti-human IgA (alpha) was purchased from KPL (Kirkegaard & Perry Laboratories, Inc. US). FITC-labelled mouse anti-human IgG1 (Fc) and FITC-labelled goat anti-human IgM were purchased from Abcam Limited (Cambridgeshire, UK). FITC-labelled rabbit anti-human C3c antibody was purchased from Dako (Dako A/S, Glostrup, Denmark).

### HBsAg and HBcAg were detected in frozen and paraffin section with immunohistochemical method

Frozen slices from biopsies of the 329 cases were kept in a low-temperature freezer. Monoclonal anti-human-HBsAg, anti-human-HBcAg and goat anti-mouse antibodies were purchased from Zhongshan Goldenbridge Biotechnology Co., Ltd. HBsAg and HBcAg were detected with immunohistochemical method in frozen and paraffin sections.

### The renal function staging, urine and renal parameters

The renal function was staged as CKD I-V according to the American standard stages of renal function [12]. The staging is based on the degree to which glomerular filtration rate (GFR) is reduced. The value of the GFR was calculated, adjusting for the creatinine level, age and body weight. The higher the level of GFR was, the more effectivity for the kidney can remove toxins. The GFR was divided into five categories: I: normal or increased GFR with renal damage, GFR > 90; II: mild decrease in the GFR with renal damage, GFR: 60–89; III: moderate decrease in the GFR, GFR: 30–59; IV: severe decrease in the GFR, GFR: 15–29; and V: renal failure, GFR < 15 or dialysis. Changes in routinely determined urine and renal parameters including the urine protein, blood urea nitrogen (BUN) and serum creatinine (Scr) levels were documented. The concentration of protein in the urine was quantitatively detected using the sulphosalicylic acid method. The level of urine protein was graded semiquantitatively on a negative to 4+ scales. The serum HBV markers and pathological subtypes of patients with high BUN and Scr levels were analysed.

Ethics approval was obtained from the ethical committee of Harbin Medical University, China.

### Statistical analysis

Statistical analysis was performed using SAS version 9.1 software (SAS Institute Inc., Cary, NC, USA). Comparisons of categorical data were performed using the chi-square test, with *P* < 0.05 being considered statistically significant.

## Results

### The relationship between different serum markers for HBV in 329 cases and the clinical and pathological typing of HBV-GN

Among the 329 cases of HBV-GN, there were many types of primary glomerulonephritis; however, MsPGN was the most common type (82/329, 24.9%, *P < 0.05*). The second most common type of HBV-GN was MPGN (64/329, 19.5%) followed by IgA nephropathy (IgAN). But IgAN (669/3136, 21.3%), MN (650/3136, 20.7%), GML (501/3136, 16%) and MsPGN (470/3136, 15.2%) were the major types in the negative control (Table 1).

**Table 1:** Histological diagnosis of HBV-positive patients (N = 329)

**Histological diagnosis**	**HBsAg positive in serum Number (%)**	**HBsAg negative in serum Number (%)**
MsPGN	82 (24.9*)	476 (15.2)
MPGN	64 (19.5)	254 (8.1)
MN	38 (11.6)	650 (20.7)
MCG	31 (9.4)	296 (9.4)
GML	30 (9.1)	501 (16.0)
IgAN	51 (15.5)	669 (21.3)
other	33 (10)	290 (9.24)
Total	329	3136

*P < 0.05.

Abbreviations: MsPGN, Mesangioproliferative glomerulonephritis; MPGN, Membranoproliferative glomerulonephritis; MN, Membranous Nephropathy; MCG, Minimal change glomerulopathy; GML, Glomerular minor lesion; IgAN, IgA Nephropathy.

Different HBV-infection markers in serum are used to judge the severity of an infection and reflect the replication of HBV in clinical practice (Table 2). The frequency of the detection of HBsAg, HBeAg and anti-HBc positivity was 48.6% (*P < 0.0*5), and 27.1% of the 329 cases were HBsAg, anti-HBe and anti-HBc positive (*P < 0.0*5). These results indicate that the damage to the glomeruli was severe when patients had serious HBV infections.

**Table 2:** Clinical significance of HBV-positive patient serum (N = 329)

**HBV-infectious markers in serum**					**Number (%)**	**Clinical significance**
**HBsAg**	**HBsAb**	**HBeAg**	**HBeAb**	**HBcAb**		
+	−	+	−	+	160 (48.6) *	acute, chronic type B hepatitis, highly contagious
+	−	−	+	+	89 (27.1) *	acute, chronic type B hepatitis, contagious
+	−	−	−	+	47 (14.3)	acute, chronic type B hepatitis, contagious
+	−	−	−	−	33 (10)	acute infection, incubation at a later period

*P < 0.05.

**Figure 1:**
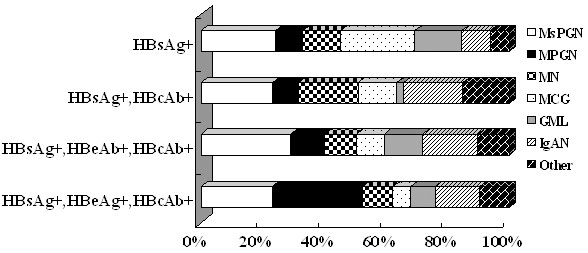
**The relationship between hepatitis serum markers and the pathological type of HBV-GN of 329 cases.** The pathological type of patients positive for HBsAg, HBeAg and Anti-HBc was most frequently MPGN (33.6%, *p < 0.05*), followed by MsPGN (24.1%, *p < 0.05*). MsPGN:Mesangioproliferative glomerulonephritis; MPGN: Membranoproliferative glomerulonephritis; MN: Membranous Nephropathy; MCG: Minimal change glomerulopathy; GML: Glomerular minor lesion; IgAN: IgA Nephropathy

The most common pathological type of patients positive for HBsAg, HBeAg and anti-HBc was MPGN (47/160, 29.4%, *P* < 0.05); MsPGN was observed in patients who were HBsAg, HBeAg and anti-HBc positive and in those who were HBsAg, anti-HBe and anti-HBc positive 37/160 (23.1%, *P* < 0.05); and 26/89 (29.2%, *P* < 0.05), respectively (Table 3, Figure 1).

**Table 3:** The relationship between serum HBV-infectious markers in 329 cases and the pathological subtype of HBV-GN

**HBV-infectious markers in serum**	**N**	**The pathological subtype of HBV-GN, number (%)**						
		**MsPGN**	**MPGN**	**MN**	**MCG**	**GML**	**IgAN**	**Other**
HBsAg+,HBeAg+,HBcAb+	160	37(23.1) *	47(29.4) *	16(10)	9(5.6)	13(8.1)	23(14.4)	15(9.4)
HBsAg+,HBeAb+,HBcAb+	89	26(29.2) *	10(11.2)	9(10.1)	8(9)	11(12.4)	16(18)	9(10.1)
HBsAg+,HBcAb+	47	11(23.4)	4(8.5)	9(19.2)	6(12.8)	1(2.1)	9(19.2)	7(14.9)
HBsAg+	33	8(24.2)	3(9.1)	4(12.1)	8(24.2)	5(15.2)	3(9.1)	2(6.1)

*P < 0.05.

### The characteristics of HBsAg and HBcAg deposits in the glomeruli of patients with HBV-GN

**Figure 2:**
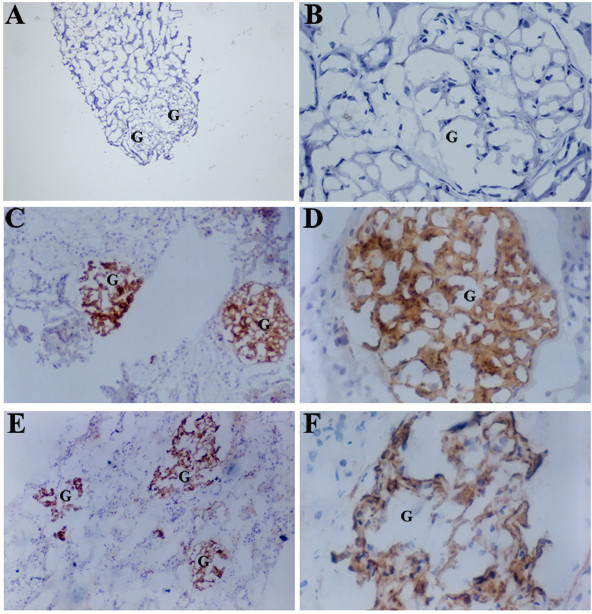
**The HBsAg and HBcAg levels in glomeruli in frozen biopsy slices.** 2**A**, 2**B** (the magnification of 2**A**): HBsAg was not present in the glomerulus and tublules in glomeruli of HBsAg-negetive cases (G indicates a glomerulus). SP × 120 and SP × 460 2**C**, 2**D** (the magnification of 2**C**): HBsAg present in glomeruli. SP × 120 and SP × 460. 2**E**, 2 **F** (the magnification of 2E): HBcAg present in the glomeruli. SP × 120 and SP × 460

HBsAg and HBcAg were detected in frozen renal biopsy sections from the 329 cases of HBV-GN. HBsAg and HBcAg were deposited in the mesangial region and in the capillary walls of glomeruli (Figures 2C, D, E, F) but not in renal tubules or in the interstitium. However, in paraffin-embedded sections, weak expression of HBsAg and HBcAg was detected mostly in the proximal convoluted tubules of glomeruli, data not shown. The present study was thus focused on the immunohistochemistry of the frozen slices. The results from the frozen sections showed a 50% expression rate of HBsAg and HBcAg in the glomeruli of patients with MPGN (*P* < 0.05) and 36.6% and 43.9% expression rates, respectively, for MsPGN (*P* < 0.05) (Table 4).

**Table 4:** The expression of HBsAg and HBcAg in glomeruli from different pathological subtypes of HBV-GN

**The pathological subtype**	**Number**	**HBsAg number (%)**		**HBcAb number (%)**	
		**+**	**-**	**+**	**-**
MsPGN	82	30(36.6)*	52(63.4)	36(43.9)*	46(56.1)
MPGN	64	32(50)*	32(50)	32(50)*	32(50)
MN	38	14(36.8)	24(63.2)	23(60.5)	15(39.5)
MCG	31	22(71)	9(29)	16(51.6)	15(48.4)
GML	30	12(40)	18(60)	13(43.3)	17(56.7)
IgAN	51	12(23.5)	39(76.5)	12(23.5)	39(76.5)
other	33	14(42.4)	19(57.6)	11(33.3)	22(66. 7)
total	329	136(41.3)	193(58.7)	143(43.5)	186(56.5)

*P < 0.05.

The relationship between the serum HBV-infection markers, age, initial symptoms, changes in routine urine and renal parameters and the stage of renal function in the 329 patients with HBV-GN.

**Figure 3:**
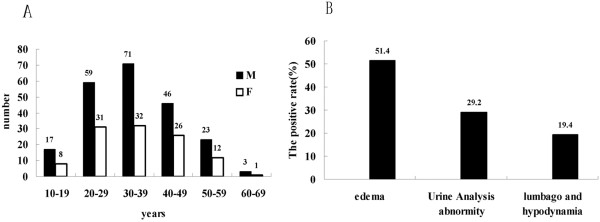
**Ages and initial symptoms of the 329 patients with HBV-GN.** 3**A**: Age distribution of the 329 cases of HBV-GN. 3**B**: Initial symptoms of the 329 cases of HBV-GN

**Figure 4:**
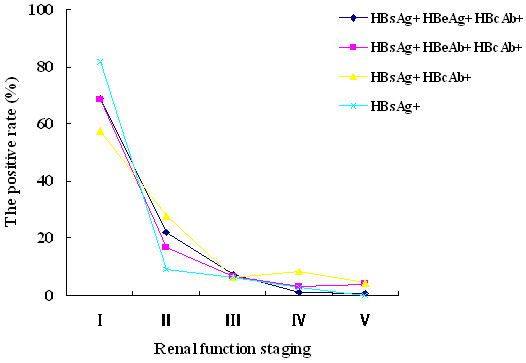
**The relationship between the renal function stage and hepatitis serum markers in the 329 cases.** The staging of renal function revealed that most patients were in stage I. The proportions of patients with glomerulus damage resulting from HBV infection were 68.39% (225/329) in stage I

**Figure 5:**
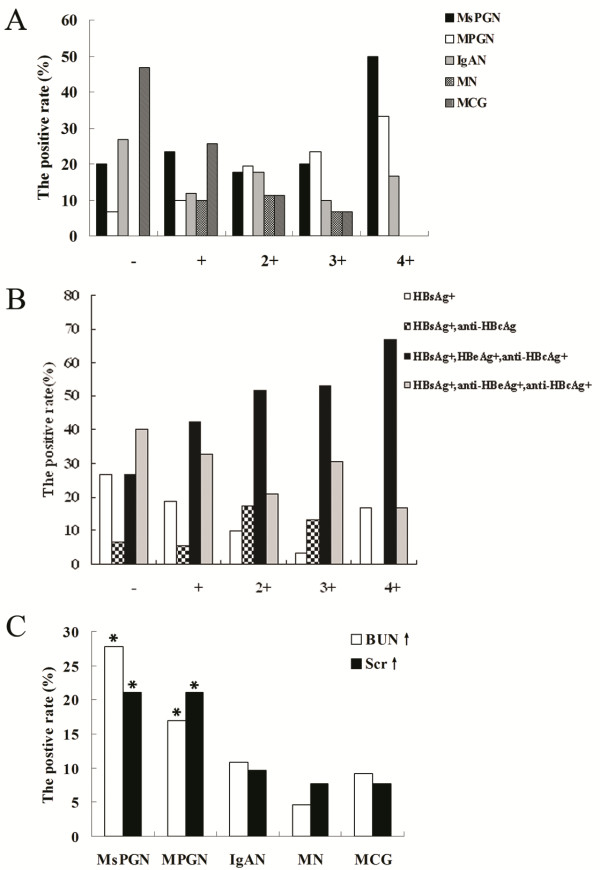
**The distribution of different pathological subtype with different urine protein and elevated BUN and Scr.** 5**A**: In the quantitatively detection of urine protein, renal function were easily damaged in patients with MPGN and MsPGN; 5**B**: Patients with HBsAg, HBeAg and Anti-HBc positive and HBsAg, Anti-HBe and Anti-HBc positive in serum were prone to leak out urine protein. 5**C**: The pathological subtype of patients with elevated BUN and Scr were MsPGN and MPGN (*P < 0.05)

Patients aged from 20 to 49 years had the highest incidence of HBV-GN, with a peak at 30 to 40 years (Figure 3A). The most common initial symptom was edema (Figure 3B). Past medical history included hepatitis lasting from several months to several decades. The relationship between the serum HBV infection markers of 329 cases and the staging of renal function revealed that most patients were in stage I. The percentage of patients with glomerular damage resulting from HBV infection was 68.4% (225/329) among stage I patients. In addition, a moderate-severe decrease in the GFR (including stages III,IVand V)to renal function failure was observed in 11.6% (38/329) (Tables 5, 6, Figure 4); however, among the 38 cases, the pathological type was MPGN in 12 cases (12/38, 31.6%, *P* < 0.05) and MsPGN in 8 cases (8/38, 21.1%, *P* < 0.05). Together, patients with MPGN and MsPGN (20/38, 52.6%, *P* < 0.05) were at a high risk of renal failure, with an unfavourable prognosis (Table 7). The quantitative detection of urine protein revealed that renal function was easily damaged in patients with MPGN and MsPGN (Figure 5A), and patients with HBsAg-, HBeAg- and anti-HBc-positive serum or HBsAg-, anti-HBe- and anti-HBc-positive serum were likely to have high levels of protein in the urine (Figure 5B). The most common pathological subtypes of patients with elevated BUN and Scr levels were MsPGN and MPGN (Figure 5C, *P* < 0.05).

**Table 5:** The relationship between serum HBV-infectious markers in 329 cases, and initial symptoms and the staging of renal function in HBV-GN

**HBV-infectious markers in serum**		**Initial symptoms number (%)**			**Renal function staging, number(%)**				
	**N**	**Edema**	**Urine abnormity**	**Lumbago and hypodynamia**	**I**	**II**	**III**	**IV**	**V**
HBsAg+,HBeAg+,HBcAb+	160	84(52.5)	49(30.6)	27(16.9)	110(68.8)	35(21.9)	12(7.5)	2(1.3)	1(0.6)
HBsAg+,HBeAb+,HBcAb+	89	45(50.6)	26(29.2)	18(20.2)	61(68.5)	15(16.9)	6(6.7)	3(3.4)	4(4.5)
HBsAg+,HBcAb+	47	24(51.1)	11(23.4)	12(25.5)	27(57.5)	13(27.7)	3(6.4)	4(8.5)	0
HBsAg+	33	16(48.5)	10(30.3)	7(21.2)	27(81.8)	3(9.1)	2(6.1)	1(3)	0
Total	329	169(51.4)	96(29.2)	64(19.5)	225(68.4)	66(20.1)	23(7)	10(3)	5(1.5)

**Table 6:** The relationship between GFR and renal function staging

**Stage**	**GFR**	**Number(%)**
I	>90	225(68.4)
II	60-89	66(20.1)
III	30-59	23(7)
IV	15-29	10(3)
V	<15	5(1.5)

**Table 7:** Moderate-severe decrease of GFR and the pathological subtype of HBV-GN

**Pathological subtype**	**Renal function staging, number (%)**			
	**III**	**IV**	**V**	**Total**
MsPGN	6	2	0	8(21.1)*
MPGN	6	5	1	12(31.6)*
MN	0	1	0	1
MCG	0	0	1	1
GML	2	0	1	3
IgAN	3	1	0	4
other	6	1	2	9
total	23	10	5	38

### The relationship between HBV antigen deposits in glomeruli and the types of Ig and complement deposits

HBsAg and HBcAg were deposited in the glomeruli of patients with HBV-GN along with the following Ig types and the following complement components. IgA deposition reached 73% (89/122), C3 deposition reached 68.9% (84/122), IgG deposition reached 78.7% (96/122), and IgM deposition reached 47.5% (58/122). The frequency of IgA deposition in patients with HBV-GN was highest in those who only expressed HBcAg in glomeruli (49/64, 76.6%), but this difference was not statistically significant (Table 8).

**Table 8:** The relationship between the HBV expression and deposit of IgA, IgM, C3, IgG

**HBsAg and HBcAg in renal tissue**	**N**	**IgA positive number (%)**	**C3 positive number (%)**	**IgG positive number (%)**	**IgM positive number (%)**
HBsAg+, HBcAg-	49	29(59.2)	29(59.2)	26(53.1)	22(44.9)
HBsAg-, HBcAg+	64	49(76.6)	44(68.8)	42(65.6)	22(34.4)
HBsAg+, HBcAg+	122	89(73)	84(68.9)	96(78.7)	58(47.5)
HBsAg-, HBcAg-	94	64(68.1)	64(68.1)	67(71.3)	43(45.7)
Total	329	231	221	231	145

## Discussion

Chronic hepatitis B and asymptomatic carriage of HBV is associated with HBV-GN [13]. Patients with HBV-GN have a high morbidity rate. In general, the clinical manifestations are not specific, and the hepatic and renal functions are normal for several years. The initial symptoms detected in medical examinations are usually proteinuria and hematuria. By this time, the renal damage is severe and irreversible. Important laboratory examinations include the evaluation of serum HBV markers, changes in renal hemodynamics and a pathological examination including a renal biopsy. These examinations are important for early diagnosis and treatment.

Some studies have investigated the relationship between HBV infection and IgA nephropathy. Lai et al. found that the morbidity due to IgA nephropathy was high in an area of high HBV prevalence. The frequency of HBsAg-positivity in IgAN serum was higher than that in healthy control serum. HBsAg, HBcAg and the corresponding immune complexes were deposited in the glomeruli. Furthermore, the deposit site was the same as that of IgA. This finding is notable because HBV and its immune complexes deposited in the glomeruli play a major role in the pathogenesis of IgAN [14]. The above mentioned studies give a hint at the close relationship between HBV infection and renal damage. HBV can involve the kidney as well as the liver, and renal function must thus be monitored in HBV-infected patients [15]. Thus, when the damage of HBV is evaluated, doctors need to focus on the change in hepatic function and to monitor the renal function index. The renal damage is related to virus replication and, more importantly, is linked to the immune response [16]. Chronic HBV infection can induce renal damage, and antigen-antibody immune complexes against HBs, HBc, or HBe together with complement components have been demonstrated to induce renal damage [17].

In the present study, the detection of HBsAg and HBcAg along with the expression of Ig types and the presence of complement components in the glomerular deposits suggests that an immune complex mechanism leads to glomerular injury. The antigen-antibody complex in renal tissues is mainly derived from blood circulation, and the circulating immune complex is detained passively in the glomeruli. The immune complex damages the kidney by activating the complement system and cytokines, which is one of the major mechanisms of the pathogenesis of HBV-GN. Jiang W [18] suggested that these autoimmunity factors may play a role in the morbidity of HBV-GN. HBcAg in renal tissue as a target antigen can initiate cytotoxic T-cell-linked immunologic injury in renal tubular epithelial cells. The pathogenesis of HBV-GN is closely related to the immune state in human, and the damage of cells after HBV infection is provoked by the immune response in the host. If a patient’s immune system is inadequate, the virus cannot be eliminated. In this state, the illness can be delayed for several years, contributing to the pathogenesis of HBV-GN.

The present study revealed that the degree of glomeruli damage was different in patients with different serum HBV infection markers. The serum HBV infection markers were predominantly HBsAg, HBeAg and anti-HBc and HBsAg, anti-HBe and anti-HBc, which were related to the severity of urine protein leakage.

The pathological type of HBV-GN as determined by renal biopsy was most frequently MsPGN, followed by MPGN. The pathological subtypes of patients with elevated BUN and Scr levels were MsPGN and MPGN. This result was different from that of previous studies that have shown that the most common histological type was MN [5-9]. The reported prevalence of HBV-GN closely parallels the geographic patterns of HBV prevalence, with marked differences in the epidemiology of HBV infection among continents and regions. Lu et al. reported that HBV S gene mutations were closely associated with HBV-GN. However, HBV serotype or genotype characteristics differ between areas; the serotype ayw and genotype A of HBV are predominant in HBV-GN in South Africa, while the endemic HBV strains are serotype adw and genotype B in south China, and serotype adr and genotype C in Korea [19]. However, the effects of these differences on the pathological subtype composition and syndrome of HBV-GN remains unclear. There have been no further worldwide reports of HBV-GN since the report by Levy and Chen [20] in the 1980s, until now. The current data based on over one hundred million people in Northeast China will provide a useful basis for further investigations into HBV-GN.

HBsAg and HBcAg were deposited in the glomeruli of 50% of the patients with the MsPGN subtype of HBV-GN, and HBsAg and HBcAg were deposited in the glomeruli in the MPGN patients of 36.6% and 43.9%, respectively. With respect to the renal function staging of the 329 cases, most patients with HBV-GN were in stage I (68.3%), and this condition was ignored by patients or doctors. Once the GFR has changed substantially, the kidneys have been irreversibly damaged. Thus, the detection of HBsAg and HBcAg in glomeruli of patients with HBV infection can predict the severity of the disease.

In general, the prognosis mainly depends on the pathological subtype. In this study, the prognosis of HBV-GN ranged from spontaneous remission to renal failure. The MPGN and MsPGN subtypes of HBV-GN represented over half (52.7%) of the 38 cases in GFR stages III-V. Patients with the minimal glomerulonephritis subtype of HBV-GN experience slow disease progression with a benign prognosis; those patients with the MsPGN subtype have a poor prognosis, and those with the MPGN subtype have the worst prognosis. Patients with MsPGN and MPGN can advance to chronic renal function failure.

Most patients with HBV-GN were male. Patients aged from 30 to 39 years old had the highest incidence of HBV-GN. Their initial symptom was edema, in agreement with another study [13]. The deposits of HBsAg, HBcAg, and simple HBcAg increased with the increase in the levels of deposits of IgA, IgG, IgM and C3 in the glomeruli. At present, the process from the onset and aggravation of HBV-GN to renal function failure is closely related to the quantity of HBsAg, HbcAg, immune globulin deposits and the pathological subtype.

## Conclusion

The study found that the most common pathological subtype in HBV-GN was MsPGN, and serological HBsAg-, HBeAg- and anti-HBc-positive patients was easy to develop MPGN. HBsAg and HBcAg were mainly deposited in the glomeruli of MsPGN and MPGN patients. In addition, cases with a moderate to severe decrease in the GFR were predominantly patients with MPGN or MsPGN. Patients who were serological HBsAg, HBeAg and anti-HBc positive or HBsAg, anti-HBe and anti-HBc positive mostly exhibited urine and renal parameter changes. Thus, clinicians should use HBV markers to judge renal disease in the early stages when it is reversible.

## Abbreviations

HBV-GN: Hepatitis B virus-associated glomerulonephritis; MsPGN: Mesangioproliferative glomerulonephritis; MPGN: Membranoproliferative glomerulonephritis; MN: Membranous nephropathy; MCG: Minimal change glomerulopathy; GML: Glomerular minor lesion; IgAN: IgA Nephropathy; GFR: Glomerular filtration rate; BUN: Blood urea nitrogen; Scr: Serum creatinine; H&E: Hematoxylin and eosin; PASM: Periodic acid-sliver methenamine.

## Authors’ contribution

LZ and HM contributed equally to this work. XH carried out the immunochemical studies. HM performed the statistical analysis. LZ summarized the data and wrote the article. XJ designed the whole experiment and submitted the article. CH, CS and FY gathered the clinical data. All authors read and approved the final manuscript.
